# Emerging Advancements in Upcycling Rice Processing Byproducts

**DOI:** 10.3390/foods15213767

**Published:** 2026-10-22

**Authors:** Sumanjot Kaur, Jaspreet Kaur, Ali Ubeyitogullari

**Affiliations:** 1 Department of Food Science, University of Arkansas, N205, 2650 N. Young Ave, Fayetteville, AR 72704, USA; 2 Department of Biological and Agricultural Engineering, University of Arkansas, Fayetteville, AR 72701, USA

**Keywords:** rice processing byproducts, rice bran, rice husk, valorization, functional foods, supercritical carbon dioxide, bioplastic, fermentation, 3D food printing

## Abstract

Rice is one of the world’s most important staple crops, but its processing generates substantial quantities of byproducts, including rice husk, bran, straw, and broken rice, which are often underutilized or disposed of through environmentally unsustainable practices. Despite being treated as waste, these byproducts are rich sources of dietary fiber, proteins, lipids, phenolic compounds, vitamins, minerals, and other bioactive constituents with significant potential for food and industrial applications. This review aims to provide a comprehensive overview of established and emerging approaches for the sustainable valorization of rice production and processing byproducts. Relevant published literature was reviewed to identify current utilization pathways, emerging technologies, associated limitations, and future opportunities. Established applications include livestock farming, biochar production, biofuel generation, brewing, and rice bran oil extraction. In addition, recent advances in value-added food applications are discussed, including supercritical carbon dioxide (SC-CO_2_)-based extraction and drying technologies, aerogel production, bioplastics, fermentation, and 3D printing. These emerging technologies enable the recovery of high-value functional compounds, improve nutritional quality, and facilitate the development of sustainable food ingredients, biodegradable packaging materials, and personalized food products. Overall, integrating advanced processing technologies into rice byproduct valorization offers a promising pathway toward a circular bioeconomy by reducing waste, enhancing resource efficiency, and creating high-value products for the food industry. Continued interdisciplinary research and industrial collaboration will be essential to accelerate commercialization and maximize the sustainable utilization of rice processing byproducts.

## 1. Introduction

Cereal crops, including rice, wheat, maize, and barley, form the backbone of global agriculture and food security [1]. Each year, millions of tons of crop residues, food processing byproducts, and unsold produce are generated as a result of agricultural processing [2,3]. These wastes, if improperly managed, can lead to environmental degradation, including soil and water pollution, greenhouse gas emissions, and loss of biodiversity. Agricultural waste represents a significant challenge and opportunity for global food security [3].

Rice (*Oryza sativa* L.) is an important food crop for billions of people, and ~11% of the world’s cultivated land is used for rice production [4]. Global rice production exceeds 700 million metric tons annually, with Asia accounting for the majority of production, particularly in countries such as China, India, Bangladesh, Indonesia, and Vietnam [4]. A significant increase has been observed in rice production since 2016, as reported by FAO, which has also resulted in an increase in byproduct production [5,6]. The utilization of these byproducts varies geographically; in major rice-producing countries, they are commonly used for animal feed, fuel, soil amendment, and other agricultural purposes. However, increasing attention worldwide is being directed toward their conversion into higher-value food, material, and bioenergy products [7]. Rice is a rich source of macro- and micronutrients, including starch, proteins, vitamin B complexes, minerals (Ca, Mg, P, Cu, Fe, Mn, Zn), and is a rich source of bioactive compounds, i.e., phenolics (caffeic acid, ferulic acid, etc.) and flavonoids (anthocyanin, proanthocyanin, isovitexin). Further, it results in high antioxidant, anti-inflammatory, and antiallergic properties, which have potential health benefits in lowering the risk of cancer, cardiovascular diseases, and heart diseases [8,9].

The commercial processing of rice kernels has different milling processes: one-step, two-step, and multi-step milling [10]. Dhankhar and Hissar [11] explain the two-step milling process for husk and bran separation, such as cleaning, dehulling, polishing, grading, and sorting. In this process, the husk and bran are removed in two steps, mainly dehulling and polishing, respectively. During dehulling, the outer layer, the husk, is removed using roll or abrasive hullers, depending on desired milling efficiency. Similarly, rice bran layers are separated through friction or abrasion to obtain a desired smooth rice grain surface [10,11,12]. Additionally, the commercial processing of rice results in high milling loss; however, advanced processing approaches, such as parboiling and fortification, were adopted to increase the bioavailability of nutrients and minimize milling losses [13].

The major byproducts produced during commercial processing still contain high nutritional value. Rice husk (~20% of total grain weight) is the outer protective covering of the rice grain, and alone has more than 100 million metric tons produced annually [14]. It contains high-value bioactive compounds, including phenolics and flavonoids. Important phenolic groups include gallic acid, pyrogallol, caffeic acid, and flavonoids such as apigenin, rutin, and coumaric acid, which provide potential health benefits [11,12,15,16,17]. Until now, rice husk has mainly been used for carbonization and biofuel production [14,18,19]. Rice husk has limited commercial applications due to its undesirable properties like high lignin content, high silica content, and high ash content, resulting in the production of CO_2_, NO_2_, methyl chloride, and other gases when it is disposed of in an open environment [16,20,21,22].

Furthermore, rice bran (~8–10% of the total grain weight) is the outer layer of the rice grain and is highly nutritious, containing a wide range of beneficial compounds, including dietary fiber, proteins, essential fatty acids, vitamins (e.g., B vitamins and vitamin E), minerals (e.g., potassium, magnesium, and zinc), and antioxidants (e.g., gamma-oryzanol and tocotrienols) [23,24]. The nutritional composition of rice bran can vary depending on factors like rice variety and processing methods [15]. It is also rich in antioxidants, which prevent the oxidative reactions of lipids and preserve foods from further deterioration, and has been associated with anti-inflammatory and anticancer effects [12,15,25,26]. Nevertheless, fibers limit their utilization in food applications due to their interactions that form complex structures, resulting in low solubility [27]. Despite the high nutritional value, the defatted rice bran is currently used for animal feed, feedstock, boiler fuel, and in limited quantities in food products (e.g., breakfast cereals) [28,29].

However, these byproducts hold significant importance due to the presence of high-value compounds and are still discarded as waste or underutilized. Recent years have seen growing interest in valorizing these byproducts due to the presence of bioactive compounds and other potential health benefits. Advancements in processing technologies have enabled the isolation of valuable compounds from these byproducts, opening new avenues for their incorporation into human food products. This shift towards more sustainable and resource-efficient food systems aligns with growing consumer demand for healthier, more environmentally conscious food options [30,31]. This review focuses on the current knowledge on rice byproduct utilization in food applications, emerging opportunities, limitations, and future outlook. Unlike previous reviews that have largely focused on the composition, nutritional properties, and conventional utilization of rice byproducts, this review emphasizes emerging value-added technologies, including SC-CO_2_ processing, aerogels, bioplastics, fermentation, and 3D food printing, to highlight new opportunities for their sustainable valorization.

## 2. Current Use of Rice Production and Processing Byproducts

Rice is one of the most widely cultivated and consumed cereal crops in the world, and its processing generates enormous volumes of residual biomass at nearly every stage of production, from field-level straw to milling-derived husk and bran [7]. For decades, these byproducts were treated largely as agricultural waste, disposed of through open burning or landfilling, practices that contribute to air pollution, greenhouse gas emissions, and loss of potentially valuable biomass. In recent years, however, rice processing byproducts have increasingly been recognized as underutilized feedstocks with substantial economic and environmental value [32,33]. This section reviews four well-established pathways for upcycling rice byproducts: conversion to biochar, use as biofuel feedstock, application in brewing, and extraction of rice bran oil. Table 1 summarizes representative recent studies across these pathways.

### 2.1. Livestock Farming

Rice straw serves as a primary coarse roughage for ruminants, fulfilling 32% to 37% of the total digestible nutrients required by livestock populations, particularly during dry-season green fodder scarcities [44]. Although raw rice straw contains low crude protein (3% to 7%) and high silica and fiber content, physical processing (such as mechanical chopping and densified complete feed block formulation) and chemical ammoniation using urea effectively break lignocellulose bonds, thereby enhancing dry matter intake, organic matter digestibility, and milk yields [45]. Beyond roughages, high-energy milling fractions like rice polish, which provides 11% crude protein and an energy density of 3250 kcal/kg, and rice bran are widely incorporated into commercial rations, with rice bran comprising up to 30% of feed formulations for dairy cattle and buffaloes [44]. Furthermore, dietary fiber and bioactive constituents in rice bran exhibit prebiotic-like properties that selectively modulate gastrointestinal microbiota (e.g., increasing beneficial taxa such as *Firmicutes* and *Lactobacillus*) and reduce the colonization of enteric pathogens like *Salmonella* in poultry [10]. Additionally, coarse rice husks are extensively utilized as bedding and litter material in poultry housing without adversely affecting bird growth performance, while energy-dense broken rice serves as a cost-effective substitute for maize in poultry starter diets and companion animal feeds [10].

### 2.2. Biochar

Rice husk accounts for roughly 20% of the weight of harvested rice and is generated in quantities exceeding 100 million metric tons annually worldwide [14], making it one of the most abundant agro-industrial residues available for thermochemical valorization [36,46]. Rich in cellulose, lignin, and amorphous silica, rice husk represents an attractive feedstock for the production of value-added carbonaceous materials. However, conventional disposal practices, including open burning and landfilling, contribute to environmental pollution, greenhouse gas emissions, and land-use challenges. Consequently, the conversion of rice husk into biochar through pyrolysis has emerged as one of the most extensively investigated and sustainable upcycling pathways for rice-processing residues [46].

Rice husk biochar (RHB) is produced by heating husk in low-oxygen conditions, and its physicochemical properties, including surface area, porosity, and functional group density, can be tailored by adjusting pyrolysis temperature, heating rate, and carrier gas flow, or through subsequent physical, chemical, or biological modification [34,47]. Due to these tunable properties, RHB has a wide range of applications, two of the most dominant in agriculture and environmental remediation. As a soil amendment and carrier for slow-release fertilizers, RHB improves nutrient-use efficiency, immobilizes heavy metals such as cadmium, enhances soil health, and supplies plant-available silicon to support crop productivity [34]. It is also widely used as a low-cost adsorbent for soil and water remediation due to its ability to remove heavy metals and organic pollutants. However, its adsorption performance may decline in the presence of competing metal ions unless the biochar is surface-modified [46].

Beyond static-bed pyrolysis, recent engineering efforts have focused on scaling RHB production through continuous pyrolysis reactors capable of achieving biochar yields of up to roughly 45% and thermal efficiencies exceeding 70%, with carbon sequestration on the order of 1.8–2.2 kg CO_2_-equivalent per kilogram of biochar produced, positioning industrial-scale RHB production as a viable component of carbon-finance and circular bioeconomy strategies [36]. Overall, rice husk biochar has evolved from a waste disposal solution into a valuable resource for sustainable agriculture and environmental remediation.

### 2.3. Biofuel

Rice straw, husk, and bran are widely utilized as feedstocks for biofuel production through conversion pathways, including bioethanol, biogas, biodiesel, and bio-oil [48]. Rice straw, generated at an estimated residue-to-product ratio of 1.70 kg of straw per kg of grain, is the largest byproduct generated after rice harvesting [37]. It is a lignocellulosic biomass rich in cellulose and hemicellulose, making it an attractive feedstock for second-generation bioethanol production [37,49]. To overcome its recalcitrant structure, rice straw typically undergoes pretreatment methods such as steam explosion, alkaline treatment, ionic-liquid treatment, or microwave-assisted ammoniation before enzymatic hydrolysis and fermentation [37]. Integrated biorefinery approaches have further shown that rice straw can be co-processed to simultaneously yield bioethanol alongside biopesticide and biofertilizer products, improving the overall economics of straw valorization [38].

Rice straw is also a proven feedstock for biogas production through anaerobic microbial digestion [39,50]. Its high cellulose content, low cost, and wide availability make it attractive for commercial-scale biogas generation, though its high carbon-to-nitrogen ratio can limit process stability when digested alone. To address this, co-digestion with de-oiled rice bran, a nitrogen-rich byproduct of the rice milling industry, has been used to improve process stability and methane yield. In a semi-continuous digester study, rice straw and de-oiled rice bran co-digestion achieved a specific methane yield of approximately 0.142 L/g total solids (0.169 L/g volatile solids), with roughly 40% volatile solids removal at optimal loading conditions [39]. Rice husk has also been widely investigated as a feedstock for bioethanol production through lignocellulosic pretreatment followed by enzymatic hydrolysis and fermentation [51,52]. In addition, it has been explored for biodiesel production, serving as both a feedstock and a source of heterogeneous catalysts, with studies reporting efficient biodiesel yields (97%) under relatively mild reaction conditions (60–70 °C) and shorter processing times (30 min) than conventional methods (1–12 h) [51,53].

Overall, the global biofuel market based on agricultural residues, including rice residues, is valued in the hundreds of billions of dollars, highlighting bioenergy as one of the most commercially significant pathways for valorizing lignocellulosic rice byproducts [48].

### 2.4. Brewing

The brewing industry is a long-established, large-scale user of rice processing byproducts, particularly broken rice kernels, bran, and other milling fractions that are unsuitable for direct table consumption [10,40]. Brewer’s rice is a byproduct of the rice milling industry, consisting of broken or downgraded rice kernels generated during the polishing process. These lower-grade fractions are typically sold at a reduced cost and are widely used in brewing and other industrial applications [40].

Rice is widely used as a brewing adjunct, serving as an alternative starch source to partially replace malted barley due to its neutral flavor, low lipid content, gluten-free nature, and lower cost [40,54]. In addition to lowering ingredient costs, rice adjuncts improve wort clarity and mashing conditions and support high-gravity brewing, while providing polyphenols that have been associated with increased antioxidant activity in the finished beer [40]. Rice is naturally gluten-free, making rice-based beers a suitable option for consumers with celiac disease or gluten sensitivity. However, its low enzymatic activity and limited free amino nitrogen content often require supplementation with commercial enzymes during the mashing process [40,43].

The rice fraction used in brewing influences both processing performance and beer quality. Compared with polished rice, brown rice retains its bran layer and contains higher levels of protein, lipids, and minerals, resulting in greater free amino nitrogen in the wort and beers with higher antioxidant capacity, although it also produces noticeable changes in color and flavor [41]. Research efforts are increasingly directed toward evaluating the brewing potential of different rice cultivars, designing heat-tolerant enzyme systems to improve rice starch utilization without specialized cooking processes, and developing rice varieties with enhanced endogenous enzyme activity to support all-rice-malt brewing [40,42].

### 2.5. Rice Bran Oil

Rice bran oil (RBO) is one of the most valuable products derived from rice milling byproducts, offering an effective approach to valorizing rice bran. Rice bran contains approximately 15–25% oil. The oil’s nutritional value is primarily attributed to its high concentration of unsaponifiable bioactive compounds [30]. Crude RBO contains approximately 15 mg/g of γ-oryzanol, a unique mixture of ferulic acid esters that provides antioxidant and anti-inflammatory properties [55]. Furthermore, RBO is a source of tocotrienols (136.7 to 1080.1 mg/kg), significantly higher than in corn oil (up to 75.5 mg/kg) or soybean oil (up to 26.4 mg/kg). It also contains phytosterols and squalene, which provide superior radical-scavenging properties compared to many conventional vegetable oils [56]. RBO contains saturated (SFAs), monounsaturated (MUFAs), and polyunsaturated fatty acids (PUFAs) present in a ratio of approximately 0.6:1.1:1. Specifically, it contains about 47% MUFA, 33% PUFA, and 20% SFA. Its primary unsaturated components are oleic acid (32.4–43.4%) and linoleic acid (28.0–53.4%). This unique balance, particularly the nearly 1:1 ratio of oleic to linoleic acid, ensures high thermal stability, making RBO an advanced cooking oil that remains stable during frying and high-temperature processing [24,30,56,57]. A critical aspect of RBO utilization involves selecting optimal refining methods to manage high free fatty acid content while retaining these bioactive nutrients. Physical refining is widely considered superior to chemical refining, as it limits γ-oryzanol loss to below 10%, whereas chemical processes can result in losses of 80–90% [6,24,30,58]. Beyond conventional edible oil applications, the high wax ester content of RBO has attracted interest in producing oleogels, in which wax crystals form a three-dimensional network that structures liquid oil. These oleogels have considerable potential as healthier replacements for saturated and trans fats in processed foods [59,60].

The favorable physicochemical properties of RBO, including its high smoke point (230–250 °C), mild flavor, and excellent oxidative stability, make it particularly suitable for high-temperature cooking applications [56]. In addition to its use, RBO is incorporated into margarines, bakery products, salad dressings, dairy alternatives, and nutraceutical formulations [55,58,60]. Rice bran wax, obtained during the refining process, is also utilized as a natural food-grade coating and structuring agent. Despite its considerable potential, the utilization of RBO is constrained by the rapid deterioration of fresh rice bran and the high costs associated with stabilization and advanced extraction technologies [61]. Nevertheless, improvements in stabilization methods and green extraction processes are expected to enhance bioactive compound retention and further expand the application of rice bran oil as a sustainable, value-added food ingredient.

## 3. Emerging Applications of Rice Processing Byproducts

Recent advances in processing and material technologies have created new opportunities for converting rice processing byproducts into higher-value products beyond their conventional uses. These emerging applications focus on the recovery and transformation of valuable components from rice bran, husk, and broken rice into functional food ingredients, bioactive compounds, sustainable packaging materials, and customized food products. Technologies such as SC-CO_2_ extraction and drying, aerogel production, bioplastics, fermentation, and 3D food printing offer advantages but also differ in their processing requirements. Table 2 provides a comparative overview of these emerging approaches, their major applications, and advantages.

### 3.1. Novel Extraction and Drying Based on Supercritical Carbon Dioxide (SC-CO_2_)

SC-CO_2_ technology is an advanced and environmentally friendly technique that utilizes CO_2_ in its supercritical state for wide applications across different industries [2]. CO_2_ is an FDA-approved, environmentally friendly, inexpensive, and safe solvent with mild critical values (31.1 °C and 7.38 MPa). As the temperature and pressure are raised above the critical values, CO_2_ exhibits a fluid state with properties of both gas and liquid, including diffusivity, density, and viscosity. SC-CO_2_ can act as a medium enabling chemical transformations under controlled conditions [64,72,73,74]. Fundamentally, SC-CO_2_ processing follows the principles of phase behavior, differential solubility, mass transfer, and thermodynamic equilibrium [75]. The solubility of target compounds in the supercritical fluid must be balanced with efficient transport across phase boundaries, which is influenced by parameters such as fluid flow rate, solute concentration, type of co-solvent, co-solvent concentration, pressure, temperature, and particle size of the feed material [2,73].

#### 3.1.1. Extraction

In terms of extraction, SC-CO_2_ has emerged as one of the most promising green technologies for the valorization of rice processing byproducts owing to its high extraction efficiency, tunable selectivity, and reduced environmental impact compared with conventional solvent-based extraction methods [2,73]. SC-CO_2_ has been successfully applied for lipid extraction from rice bran [62] and the recovery of high-value bioactive compounds, particularly phenolic compounds and flavonoids, from rice husk [2]. The extraction performance is strongly influenced by pressure and temperature, which determine the density, diffusivity, and solvating power of the supercritical fluid. Generally, increasing pressure enhances CO_2_ density and solvent strength, thereby improving the solubility of target compounds; however, excessive pressure may reduce diffusivity and mass transfer efficiency, limiting extraction yields. Therefore, extraction behavior is governed by the crossover-pressure phenomenon, where the dominant factor controlling solubility shifts from solvent density to solute vapor pressure. For example, Kaur and Ubeyitogullari [2] reported higher total phenolic and flavonoid yields from rice husk at 30 MPa than at 40 MPa, suggesting that improved matrix penetration and mass transfer at lower fluid density can outweigh the benefits of increased solvent strength. Previous studies have demonstrated the sequential recovery of lipids, volatile compounds, phenolics, and flavonoids through controlled changes in extraction parameters, while the addition of co-solvents such as ethanol further enhances the extraction of polar compounds [73,76]. Compared with conventional extraction techniques, including Soxhlet extraction, liquid–liquid extraction, and solid–liquid extraction using organic solvents such as hexane, methanol, acetone, and chloroform, SC-CO_2_ offers significant environmental, safety, and product-quality advantages [77,78]. Traditional methods typically require large volumes of organic solvents, prolonged extraction times, energy-intensive solvent recovery, and additional purification steps, while also posing concerns regarding solvent residues, toxicity, flammability, and oxidative degradation of sensitive compounds [23,79]. In contrast, SC-CO_2_ utilizes a non-toxic, non-flammable, FDA-approved solvent that leaves no residual solvent in the final product and operates under oxygen-limited conditions, thereby preserving the quality of thermolabile bioactive compounds. Furthermore, the closed-loop nature of SC-CO_2_ systems enables approximately 95–99% recycling of CO_2_, resulting in minimal atmospheric emissions and a substantially reduced environmental footprint [80]. Although the relatively high capital investment and demanding operating conditions of SC-CO_2_ systems pose important challenges to large-scale commercialization, particularly compared with conventional solvent extraction technologies, the environmental benefits, superior selectivity, and ability to produce high-purity extracts continue to drive research and industrial interest in this technology [81].

#### 3.1.2. Aerogel

**Figure 1:**
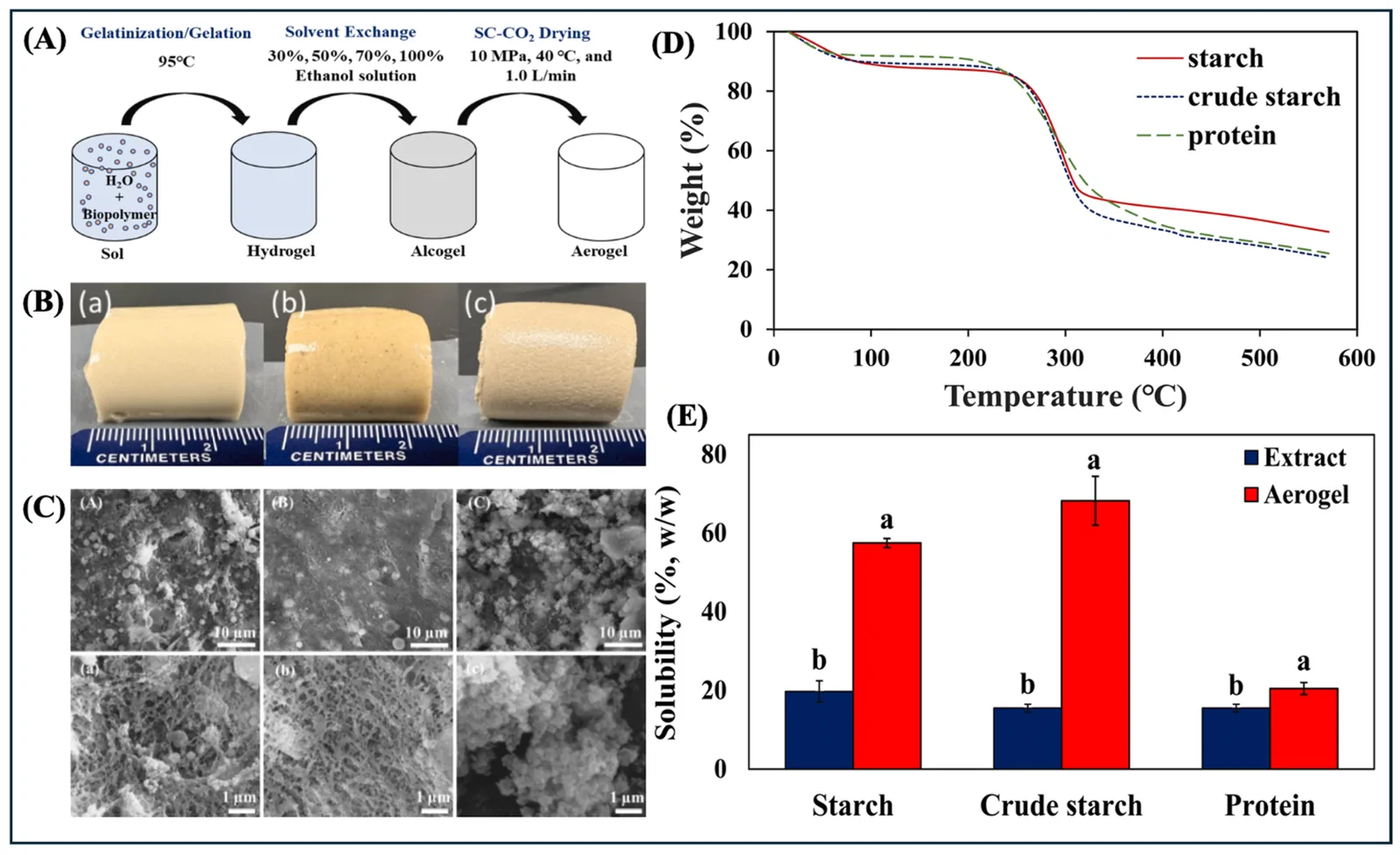
(**A**) A diagram of aerogel formation steps, (**B**) pictures of rice bran-based starch (**a**), crude starch (**b**), and protein (**c**) hydrogels, (**C**) SEM images of starch (**A**,**a**), crude starch (**B**,**b**), and protein (**C**,**c**) aerogels, (**D**) thermal stability of aerogels, and (**E**) water solubility of aerogels. Modified from Kaur, et al. [64] and Kaur and Ubeyitogullari [63].

In terms of aerogel production, SC-CO_2_ drying has emerged as one of the most effective approaches for preserving the highly porous three-dimensional structure of biopolymer-based gels and producing high-quality aerogels with superior physicochemical properties such as thermal stability and solubility (Figure 1). Aerogels are nanoporous materials characterized by exceptionally high surface area, low density, high porosity, and low thermal conductivity, making them attractive for applications in thermal insulation, adsorption, biomedical engineering, drug delivery, and functional food systems [82]. Rice processing byproducts represent a promising and underutilized feedstock for aerogel production because they are rich in cellulose, starch, proteins, silica, and lignin. The fabrication of aerogels generally involves hydrogel formation, solvent exchange, and drying, with the drying stage being the most critical step in determining the final structural properties of the aerogel. During drying, the liquid phase must be removed without collapsing the gel network, as capillary stresses generated at the liquid-vapor interface can significantly reduce porosity and surface area. To address this challenge, SC-CO_2_ drying is employed because it eliminates the liquid–gas interface and minimizes surface tension effects, thereby preserving the nanoporous structure and reducing shrinkage. Prior to drying, solvent exchange with ethanol is commonly performed due to its low surface tension and complete miscibility with SC-CO_2_, which facilitates efficient solvent removal while maintaining structural integrity [64,82]. Compared with conventional drying methods such as air drying and freeze drying, SC-CO_2_ drying offers substantial advantages in preserving pore architecture and minimizing structural deformation. Air drying is simple and inexpensive but often causes severe shrinkage and pore collapse due to high capillary forces generated during solvent evaporation [82]. Freeze drying reduces capillary stresses through sublimation; however, ice crystal formation, prolonged processing times, and high energy consumption can adversely affect pore morphology and mechanical stability [83]. Consequently, aerogels produced by conventional drying methods generally exhibit lower surface area, reduced porosity, and less uniform microstructures than those produced through SC-CO_2_ drying [84]. The advantages of SC-CO_2_ drying have enabled the development of protein-, starch-, and cellulose-based aerogels from agricultural materials, including rice bran-derived proteins and starches, and rice husk cellulose, which have demonstrated high surface areas of 36–47 m^2^/g, porosities (>83%), and low densities (<0.3 g/cm^3^). Also, at 15% (*w*/*w*) concentration, these aerogels exhibited interconnected porous structures and high digestibility (73–86%), further supporting their potential as carriers for bioactive compounds [64,65]. The superior ability of SC-CO_2_ drying to preserve nanoporous structures, combined with the growing demand for sustainable and high-performance biomaterials, positions this technology as a promising platform for converting rice processing byproducts into value-added aerogel-based products for food, pharmaceutical, biomedical, and environmental applications.

Similarly, rice husk has been upcycled into cellulose nanofiber (CNF) aerogels through cellulose extraction, nanofibrillation, and SC-CO_2_ drying. Rice husk is particularly attractive as a cellulose source because it contains approximately 25–48% cellulose, although its lignin and silica contents necessitate delignification and purification prior to aerogel formation. Among different CNF preparation approaches, acid hydrolysis coupled with ultrasonication produced aerogels with a high surface area (~231 m^2^/g), pore volume (1.41 cm^3^/g), and pore size (~29 nm), while ultrasonicated samples achieved ~87% methylene blue adsorption, demonstrating the potential of rice husk-derived aerogels for adsorption and wastewater-treatment applications [65]. Therefore, these findings support the use of food-processing byproducts as low-cost cellulose sources for producing high-value porous materials and advancing waste valorization within a circular bioeconomy [85].

### 3.2. Bioplastic

Bioplastics comprise a broad group of materials that may be bio-based, biodegradable, or both. Bio-based plastics are derived wholly or partly from renewable biological resources, whereas biodegradable plastics are defined by their ability to undergo microbial degradation under specified environmental conditions and may be derived from either renewable or fossil-based resources. Compostable plastics are a subset of biodegradable plastics that biodegrade and disintegrate under defined composting conditions [86]. These materials serve as a sustainable alternative to conventional petroleum-derived plastics, which contribute to global pollution through the accumulation of persistent waste and the release of microplastics into the ecosystem [66,87]. Rice husk and rice bran have been proposed for generating bioplastics through fundamentally different mechanisms. Rice husk contains high cellulose, lignin, and silica content and is used primarily as a reinforcing filler within polymer matrices such as polylactic acid, polyhydroxyalkanoates, or thermoplastic starch, improving tensile strength and modifying biodegradation rates [67]. Husk ash can also be processed into amorphous nanosilica, which is incorporated as a filler to enhance thermal stability and barrier properties [87,88]. On the other hand, defatted rice bran can serve as a filler similar to husk, but its starch, protein, and wax fractions can also function as the actual structural matrix of the bioplastic itself, rather than only reinforcing one [89,90]. Research shows that refining the raw material by extracting rice bran oil (lipids) significantly improves the viscoelastic moduli and tensile behavior of the resulting bioplastic while decreasing water absorption [90]. Furthermore, optimizing injection molding conditions, such as using a mold temperature of 150 °C, can lead to the development of stiffer and more resistant food-service items [89].

Further, rice husk improves the properties of bioplastic films by incorporating up to 3 wt.% rice husk biogenic silica into cellulose biopolymer matrices, creating a dense network that improves overall film performance, with maximum tensile strength from 311 kN/m^2^ up to 1347 kN/m^2^ at 2 wt.% silica loading, while lowering film moisture content from 10.5% down to 3.4%, reducing water vapor permeability, and decreasing water mass loss from 63.8% to 46.1% [87]. Bioactive molecules like arabinoxylans and phenolic compounds extracted from rice husk can be incorporated into bioplastic films to impart antioxidant properties, achieving an antioxidant capacity more than 10-fold higher than control films [91]. Additionally, the integration of silica from rice husk ash into starch-based bioplastics increases the material’s thickness and density while reducing moisture sensitivity, thereby preventing mold formation and microbial activation [87,88]. Advanced processing of rice processing residues further enhances their utility for specialized food packaging. The extraction of nanocellulose from rice husks to reinforce gelatin-based films improves their compactness and water resistance for use as sustainable food wraps [87,92]. Valsalan, et al. [92] developed gelatin films strengthened with rice husk-derived nanocellulose, nano-MgO, and neem oil that exhibited enhanced food-packaging performance. Incorporation of nanocellulose improved barrier properties, nano-MgO enhanced thermal stability and biodegradability, while neem oil provided strong antibacterial activity against *E. coli* and improved flexibility (~100–120% elongation). Further, Mohammad Azmin, et al. [88] developed rice husk-sweet potato peel bioplastic optimized by response surface methodology with 1.87 mm thickness, 0.196 g/mL density, and 31.22% moisture content, demonstrating its potential as a sustainable food-packaging material. These innovations demonstrate the potential of using rice processing byproducts to develop functional, active, and eco-friendly packaging that contributes to a circular bioeconomy.

### 3.3. Fermentation

Fermentation is a metabolic process where microorganisms break down complex organic substrates under controlled aerobic or anaerobic conditions to produce value-added products. For rice processing byproducts like bran, husk, and broken grains, solid-state fermentation (SSF) is particularly effective because it allows microbes to grow on solid particles with minimal moisture, providing an economically viable and environmentally sustainable method for bioconversion [93,94]. Major components of rice byproducts utilized or transformed during fermentation include starch and other carbohydrates, proteins, fiber-associated polysaccharides, and amino acids such as glutamate, resulting in products such as organic acids, free amino acids and peptides, GABA, enzymes, and enriched or released bioactive compounds. This biotechnological tool is used across food, health, and cosmetic industries to improve the functional and nutritional profiles of underutilized agricultural residues [95,96]. By utilizing various fungi and bacteria, fermentation transforms these residues into high-value functional ingredients, addressing environmental concerns related to waste disposal while meeting growing consumer demands for health-promoting foods [93,96].

One of the primary value-addition benefits of fermentation is the nutritional upgrading of rice processing byproducts, particularly in terms of protein and mineral availability. Fermentation can also improve the quality of rice bran proteins through microbial and enzymatic hydrolysis, which can increase protein solubility and digestibility, release peptides and amino acids, and reduce antinutritional factors that limit nutrient utilization. Islam, et al. [97] investigated the fermentation of rice bran using *Saccharomyces cerevisiae*, which revealed significant quantitative improvements, including a 23.1% increase in crude protein content. The study also revealed that the process reduced crude fiber by 20.1% and significantly enhanced mineral bioavailability, with available phosphorus increasing by 55.6% due to the degradation of anti-nutritional factors like phytates [97]. Furthermore, co-fermentation using *Lactiplantibacillus plantarum* and *Bacillus subtilis* was investigated to optimize the bacterial community and increase the concentration of essential amino acids and organic acids by catabolizing proteins and carbohydrates [68,98]. The solid-state fermentation of rice bran with *Rhizopus oryzae* revealed that the ferulic acid content increased dramatically from 33.3 μg/g to 764.7 μg/g [93]. In another study involving rice germ, precision fermentation using *Levilactobacillus brevis* BJ20 was optimized to produce gamma-aminobutyric acid (GABA), achieving production through the bioconversion of glutamate [99]. These biochemical changes are often driven by extracellular enzymes like cellulases and xylanases that break down the plant cell wall, releasing bound phenolic compounds and increasing the total phenolic content and radical scavenging activity of the substrate [96,100,101].

In the food industry, fermentation facilitates the development of innovative products such as probiotic beverages and high-purity organic acids. Rice milling byproducts can be used to produce healthy cereal-based milk analogs; for instance, a study using subcritical water extraction followed by fermentation with *Lactobacillus casei* T20 reported an increase in total phenolic compounds to 14.5 mg GAE/ml and enhanced DPPH radical scavenging activity [69]. Moreover, white rice bran is a cost-effective substrate for the industrial production of L-lactic acid, utilizing *Bacillus coagulans*, yielding 117 g/L of lactic acid and achieving a productivity of 2.79 g/L/h [102]. This provides a sustainable alternative for producing biodegradable plastics and other industrial chemicals from renewable resources [102].

### 3.4. 3D Food Printing (3DFP)

3DFP is a type of additive manufacturing technology that constructs complex, freeform geometries layer-by-layer from edible materials [70,103]. In the field of food science, 3DFP is recognized for its transformative potential, offering unique advantages such as high design precision, the ability to create intricate patterns without molds, and a reduction in material waste with a material efficiency rate of approximately 97% [104,105,106]. The technology’s primary importance lies in personalized nutrition and customization. 3DFP allows precise control over a product’s density, structure, and nutrient composition to meet the specific dietary requirements of demographic groups such as children and the elderly [105]. For instance, 3D-printed food can be designed with soft, pureed textures to assist patients with dysphagia (swallowing difficulties) [105,107]. Beyond medical applications, 3DFP supports environmental sustainability by repurposing food processing byproducts, including rice processing byproducts, into high-value functional foods, contributing to the United Nations’ Sustainable Development Goals (SDGs), with the potential to mitigate 130.5 to 525.5 Mt of greenhouse gas emissions by 2025 [105,106].

**Figure 2:**
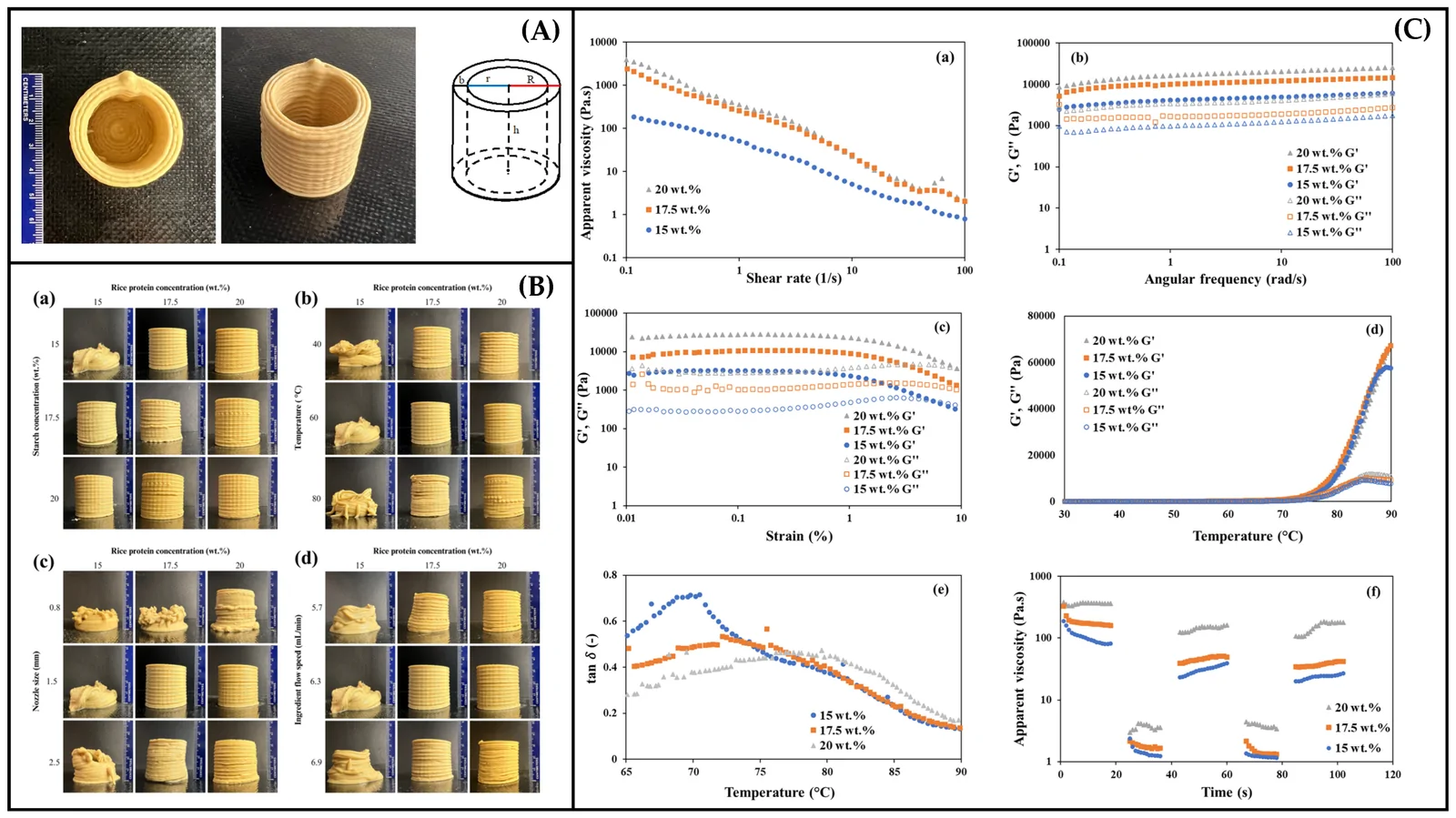
(**A**) Photographs and design of the 3D-printed object; (**B**) Photographs of (**a**) RP-S gels 3D-printed with various concentrations (printing temperature: 60 °C, nozzle size: 1.5 mm, and flow speed: 6.3 mL/min), (**b**) RP-S gels 3D-printed with constant S concentration of 15 wt.% at various RP concentrations and printing temperatures (nozzle size: 1.5 mm, and flow speed: 6.3 mL/min), (**c**) RP-S gels 3D-printed with constant S concentration of 15 wt.% at various RP concentrations and nozzle sizes (printing temperature: 60 °C, and flow speed: 6.3 mL/min), and (**d**) RP-S gels 3D-printed with constant S concentration of 15 wt.% at various RP concentrations and ingredient flow speeds (printing temperature: 60 °C, and nozzle size: 1.5 mm). (**C**) (**a**) Apparent viscosity as a function of shear rate; (**b**) storage (G′) and loss (G″) moduli as a function of angular frequency; (**c**) strain sweep curves; (**d**) temperature ramp curves; (**e**) loss tangent as a function of temperature; and (**f**) recovery tests of RP-S gels at a S concentration of 15 wt.% and RP concentrations of 15, 17.5, and 20 wt.%. RP, Rice protein; S, starch. Source: Nguyen, et al. [70].

3DFP requires food inks to possess specific rheological properties, most notably shear-thinning behavior. This non-Newtonian characteristic allows the material’s viscosity to decrease under high shear stress at the nozzle tip, facilitating smooth extrusion, followed by a rapid recovery of structural integrity (high storage modulus, G′) to support subsequent layers [70,108]. Additionally, nozzle size, concentration, flow speed, and temperature play a critical role in printability as revealed in Figure 2. For example, rice protein (RP) and starch (S) mixtures have been optimized for the alternative protein industry [70]. A study revealed that the optimal 3D printability for RP-S gels was achieved using a formulation of 17.5% rice protein and 15% starch, printed at 60 °C with a 1.5 mm nozzle and a flow speed of 6.3 mL/min [70]. Further, rice starch-based inks exhibit peak storage modulus values between 70 and 80 °C due to gelatinization, which forms a closely packed gel network suitable for maintaining shape fidelity [103]. Huang, et al. [107] revealed that physical modification of rice starch via dry heating for 45 min has been shown to produce safe, elderly-friendly foods with a hardness value of <30 g force, significantly reducing choking risks for dysphagia patients while lowering total in vitro digestibility to 63.28% for better glycemic control.

Bioactive compounds extracted from rice bran, such as black rice anthocyanins, are used to enhance the functionality and sensory appeal of 3D-printed gels [104]. Research demonstrated that encapsulation of black rice anthocyanins into starch gels significantly delayed pigment degradation, with retention levels 11.91 mg/L higher in dark and 13.06 mg/L higher in light storage compared to free anthocyanins. Texture analysis showed superior structural resistance in acidic environments [104]. Further, rice husk and rice bran were used for sustainable food packaging filaments [109,110,111]. While raw rice husk often clogs nozzles due to its fibrous nature, milling it into a finer fraction (0.125 mm) and adding 1% guar gum as a binding agent enables successful extrusion at 2100 mm/min and 300 rpm [110]. In bioplastic applications, 3D-printed rice bran/PLA composites with 20 wt.% bran content have shown promising mechanical performance, achieving a flexural strength, highlighting how agricultural waste can be transformed into wealth through advanced digital manufacturing [111]. 

## 4. Limitations of Emerging Applications from Rice Byproducts

Despite considerable progress in the valorization of rice bran and rice husk, several limitations continue to restrict the scalability, consistency, and sustainability of current and emerging processing approaches. Raw material variability is a major challenge, as the composition of rice byproducts can vary with rice variety, cultivation conditions, geographical origin, milling practices, and storage conditions [7]. Such variability can affect extraction yields, processing behavior, and the physicochemical and functional properties of the resulting ingredients and materials. In addition, the relatively low bulk density and geographically dispersed availability of rice husk and bran can increase transportation, handling, and storage requirements, potentially reducing the economic and environmental benefits of their valorization.

For rice husk in particular, its high lignin and silica contents present important processing challenges. Lignin contributes to the structural recalcitrance of the lignocellulosic matrix and can limit the accessibility and efficient isolation of cellulose and other carbohydrate fractions. Rice husk is also distinguished by its high silica content, which contributes substantially to its high ash content and can complicate processing, equipment operation, and the production of purified cellulose-based materials. Although silica itself can represent a valuable co-product when selectively recovered, its presence may be undesirable when the primary objective is the isolation of high-purity cellulose or other organic fractions [112]. Consequently, conventional husk fractionation often requires intensive pretreatment and purification procedures. These processes may involve strong alkalis, acids, bleaching agents, and other harsh chemicals for delignification, silica removal, and cellulose purification, generating chemical-intensive waste streams and increasing water and energy consumption [65]. The environmental advantages of producing value-added materials from rice husk may therefore be diminished if extensive chemical treatment is required.

Emerging technologies, including subcritical water, SC-CO_2_, and other green extraction and processing approaches, can reduce dependence on conventional organic solvents and enable more selective recovery of valuable components [7]. However, their broader implementation requires further process optimizations to overcome challenges associated with scale-up and continuous operation. Furthermore, replacing conventional processing with an emerging technology does not automatically ensure greater sustainability; the overall benefits depend on factors such as pretreatment requirements, solvent and water consumption, energy demand, product yield, co-product utilization, and waste generation. Further, the emerging technologies discussed in this review also differ considerably in technological maturity and scalability. SC-CO_2_ extraction is relatively well established and offers high selectivity, reduced solvent residues, and improved product quality, and it exists at large scales for various applications such as decaffeination and essential oil extractions. Fermentation is a comparatively accessible approach because of its established use in food processing, although optimization is required for specific rice byproducts. In contrast, rice-byproduct-derived aerogels, bioplastics, and 3D-printed foods remain largely at the laboratory or developmental stage despite their promising functional properties. Overall, these technologies involve trade-offs among processing efficiency, environmental benefits, cost, energy requirements, and scalability that should be considered when assessing their potential for industrial implementation. Future research should therefore emphasize integrated fractionation and biorefinery strategies that minimize purification and chemical inputs, accommodate natural variability in rice byproducts, recover multiple valuable fractions—including silica and lignin and consider transportation and supply-chain requirements alongside processing performance.

## 5. Conclusions and Future Directions

Rice processing generates large quantities of byproducts that have traditionally been regarded as low-value residues or agricultural waste. However, growing evidence demonstrates that rice husk, rice bran, straw, and broken rice are valuable sources of dietary fiber, proteins, lipids, phenolic compounds, phytochemicals, and other bioactive constituents that can be transformed into functional food ingredients and sustainable biomaterials. Conventional applications, including biochar production, biofuels, brewing, and rice bran oil extraction, have already established commercial value while reducing environmental burdens associated with waste disposal.

Recent technological advances have substantially expanded the potential of rice byproduct utilization. Green extraction technologies, particularly SC-CO_2_ extraction, provide efficient recovery of high-value bioactive compounds while minimizing solvent use and preserving product quality. Likewise, SC-CO_2_ drying enables the production of highly porous aerogels suitable for encapsulation and controlled delivery of bioactive compounds. Fermentation has emerged as an effective strategy for improving nutrient bioavailability, increasing phenolic content, and producing functional metabolites such as γ-aminobutyric acid (GABA) and organic acids. Furthermore, rice-derived biopolymers and nanomaterials have shown considerable promise for developing biodegradable food-packaging materials, while advances in 3D food printing provide new opportunities for incorporating rice-derived proteins, starches, and bioactive compounds into personalized and functional foods. Despite these advances, several challenges continue to limit large-scale commercialization. Variability in raw material composition due to cultivar, cultivation conditions, and processing methods affects product consistency and process standardization. Many emerging technologies remain constrained by high capital investment, energy requirements, and limited industrial-scale validation. In addition, comprehensive life-cycle assessments, techno-economic analyses, and regulatory frameworks are needed to evaluate the sustainability, safety, and commercial feasibility of novel rice byproduct-derived products. Consumer acceptance and market competitiveness will also play important roles in successful commercialization.

Future research should therefore focus on developing integrated biorefinery approaches capable of simultaneously recovering multiple high-value compounds from rice processing residues while minimizing waste generation. Optimization of green extraction technologies, fermentation processes, and advanced material fabrication techniques should be prioritized to improve process efficiency and reduce production costs. Greater emphasis should also be placed on the development of multifunctional food ingredients, active and intelligent food packaging, encapsulation systems for bioactive delivery, and personalized nutrition platforms enabled by additive manufacturing technologies. Integrating artificial intelligence, process modeling, and digital manufacturing with sustainable processing technologies may further accelerate process optimization and industrial implementation. Collectively, these advances will facilitate the transition from conventional waste management to a circular bioeconomy, positioning rice processing byproducts as sustainable resources for the next generation of functional foods, food packaging, and nutraceutical products.

## Figures and Tables

**Table 1:** Summary of recent studies on upcycling of rice husk, straw, and bran.

Byproduct	Application	Key Finding	References
Rice husk	Biochar—Slow-release fertilizer	Composite RHB fertilizers enable simultaneous slow-release nutrient delivery and cadmium immobilization in contaminated paddy soils	Tan, et al. [34]
Rice husk	Biochar—Adsorbent	Effective for heavy metal/organic pollutant removal from soil and water; performance reduced by competing metal ions unless surface-modified	Li, et al. [35]
Rice husk	Biochar—Continuous pyrolysis	Continuous reactors achieve ~45% biochar yield, >70% thermal efficiency, and 1.8–2.2 kg CO_2_-eq sequestered per kg biochar	Subbiah, et al. [36]
Rice straw	Bioethanol—2nd generation	Pretreatment (steam explosion, alkaline, ionic liquid) required to overcome lignocellulosic recalcitrance before fermentation	de Souza, et al. [37]
Rice straw	Integrated biorefinery	Co-production of bioethanol, biopesticide, and biofertilizer from a single straw feedstock	Saini, et al. [38]
Rice straw + de-oiled bran	Biogas (Anaerobic co-digestion)	~0.142 L CH_4_/g TS under optimal loading; co-digestion improves stability vs. straw alone	Jha, et al. [39]
Milled/broken rice	Brewing adjunct	Lowers cost, improves wort clarity and mashing, supports high-gravity brewing	Molligoda and Anwar [40]
Brown rice (bran-retained)	Brewing adjunct comparison	Higher FAN and antioxidant capacity vs. polished rice, with greater flavor/color impact	Wang, et al. [41]
Milled rice (Multiple cultivars)	Brewing equipment (process optimization)	Cultivar characterization + heat-stable exogenous enzymes enable milled-rice brewing without cereal-cooker infrastructure	Aitkens and Lafontaine [42]
Rice (All malt)	Gluten-free brewing	All-rice-malt beer viable for celiac-disease consumers; low FAN/diastatic power requires enzyme supplementation	Mayer, et al. [43]

**Table 2:** Emerging technologies for value-added utilization of rice processing byproducts.

Technology	Rice Byproduct	Main Product/Application	Key Advantages	Reference(s)
SC-CO_2_ extraction	Rice bran, rice husk	Oils, phenolics, flavonoids and other bioactive extracts	Low solvent residues; tunable selectivity; mild processing conditions; reduced use of conventional organic solvents	Kaur and Ubeyitogullari [2], Benito-Román, et al. [62]
Aerogels (SC-CO_2_ drying)	Rice bran-derived protein/starch, rice husk-derived cellulose	Porous aerogels for encapsulation and delivery of bioactives and other food-related applications	High porosity and surface area; reduced structural collapse during drying; potential for controlled delivery and encapsulation	Kaur and Ubeyitogullari [63], Kaur, et al. [64], Kaur, et al. [65]
Bioplastics	Rice bran, rice husk, rice straw-derived fractions	Bio-based films, composites and food-packaging materials	Valorization of fiber, cellulose and silica-rich fractions; potential functional packaging properties	Alonso-González, et al. [66], Yeboah, et al. [67]
Fermentation	Rice bran, broken rice and other nutrient-rich rice fractions	Functional ingredients and fermented products enriched in bioactive compounds/metabolites	Improve nutrient availability and bioactive compounds; relatively mild processing conditions	Hou, et al. [68], Raiesi Ardali, et al. [69]
3D food printing	Rice protein, rice starch, broken rice and rice-derived ingredient blends	Customized/personalized foods and functional food formulations	Product customization; controlled geometry and composition; potential incorporation of nutrient- and bioactive-rich byproducts	Nguyen, et al. [70], Hamad and Tayel [71]

## Data Availability

No new data were created or analyzed in this study. Data sharing is not applicable to this article.
